# A case report of aortic root repair using a pericardial autograft for type A aortic dissection

**DOI:** 10.1186/s13019-020-01356-z

**Published:** 2020-10-17

**Authors:** Yi Chang, Hongwei Guo, Xiangyang Qian, Fang Fang

**Affiliations:** Department of Surgery, Fuwai Hospital, National Center for Cardiovascular Diseases, Chinese Academy of Medical Sciences and Peking Union Medical College, 167# Beilishi Road, Beijing, 100037 China

**Keywords:** Surgical repair, Aortic dissection, Sinus of Valsalva, Aortic insufficiency, Pericardial autograft

## Abstract

**Background:**

Acute type A aortic dissection with a dissection flap extending into the sinus segment often involves the commissures and the coronary ostia. In most cases, the intimal flap must be retained in order to restore aortic valve competence and reconstruct the coronary ostia. Residual dissection flap has the potential risks of proximal bleeding and adverse effects on long-term durability. We established a novel technique to reconstruct the aortic root using a pericardial autograft and significantly reduce remnant dissection tissues.

**Case presentation:**

A 50-year-old female was admitted to our center with acute anterior chest pain and backache lasting about 10 h. Computed tomographic (CT) scans showed type A aortic dissection, with both coronary ostia being involved. Doppler echocardiography showed moderate aortic insufficiency. The dissection intimal flap was removed to the normal aorta wall near the annulus at the noncoronary sinus, leaving a 5 mm rim of intimal flap near the commissures and coronary ostia. Using a pericardial patch as a new aortic wall to reconstruct the root while preserving the aortic adventitia to fix and strengthen the new pericardial aortic wall. Ascending aorta and total arch replacement combined with frozen elephant trunk procedure was performed at the same time. The patient got an uneventful postoperative course.

**Conclusion:**

**A**ortic root repair with a pericardial autograft is a safe and effective technique to treat acute type A dissection involving the sinus. Using this technique, residual dissection tissues could be significantly reduced, which subsequently decreases the risk of proximal bleeding and hence increases long-term durability.

## Introduction

Acute type A aortic dissection with a dissection flap that extends into the sinus often results in aortic insufficiency and involvement of the coronary ostium. There are several surgical techniques to reconstruct the sinus, restore aortic valve competence, and repair the coronary ostia. Reconstruction techniques include external, internal, or intramural reinforcements with prosthetics, biologics, or autologous materials [1–4]. However, there will be residual dissected tissues at the root which could have adverse effects on the long-term durability of the aortic root. Herein, we established a novel technique to reconstruct the sinus, restore aortic valve competence, and repair the coronary ostia using pericardial autografts, so as to significantly reduce residual dissected tissues.

## Case presentation

The study protocol was approved by the Ethics Committees of the Fuwai Hospital, National Center for Cardiovascular Diseases, Chinese Academy of Medical Sciences and Peking Union Medical College, Beijing, and the participant provided written informed consent.

A 50-year-old female was admitted to our center with acute anterior chest pain and backache lasting about 10 h. Computed tomographic (CT) scans showed type A aortic dissection, with both coronary ostia being involved. Doppler echocardiography showed moderate aortic insufficiency.

A median sternotomy was performed with cannulation of the right femoral artery, right axillary artery, and right atrium and venting of the left heart from the right superior pulmonary vein.

**Fig. 1:**
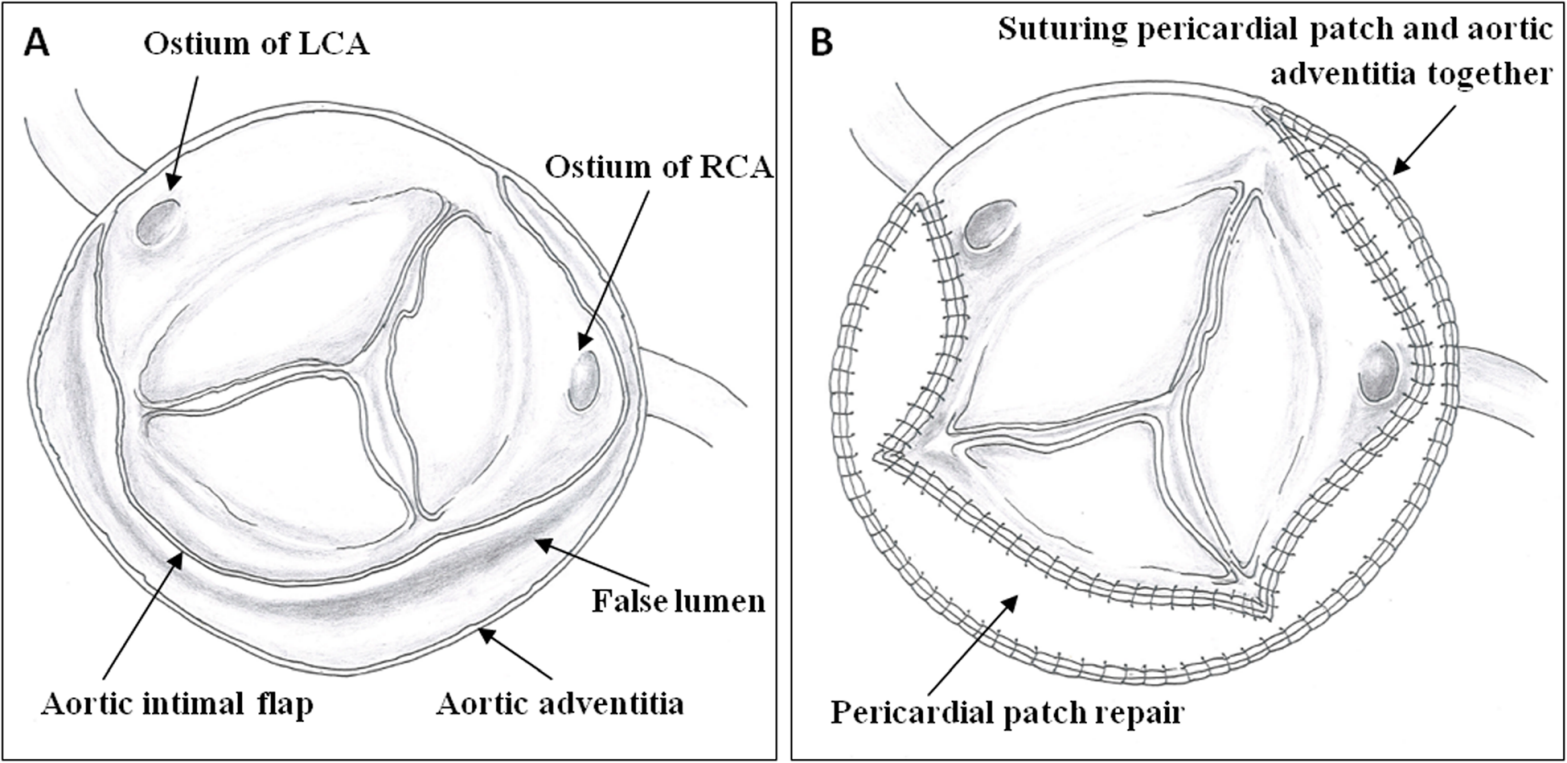
**a** Aortic dissection involving the commissures of the noncoronary-right and noncoronary-left coronary cusp, both coronary ostia, and noncoronary sinus. LCA: left coronary artery; RCA: right coronary artery. **b** After removing the dissection intimal flap, pericardial autograft patch was used to create a new aortic wall to reconstruct the sinus, coronary ostia, and resuspend the commissures. A pericardial patch was sutured to the aortic adventitia to strengthen the new aortic wall

Inspection of the aortic root showed the dissection flap extending into the sinus and involved both commissures of the noncoronary-right coronary cusp and noncoronary-left coronary cusp, both coronary ostia, and noncoronary sinus near the aortic annulus. The aortic cusps were normal (Fig. 1a).

**Fig. 2:**
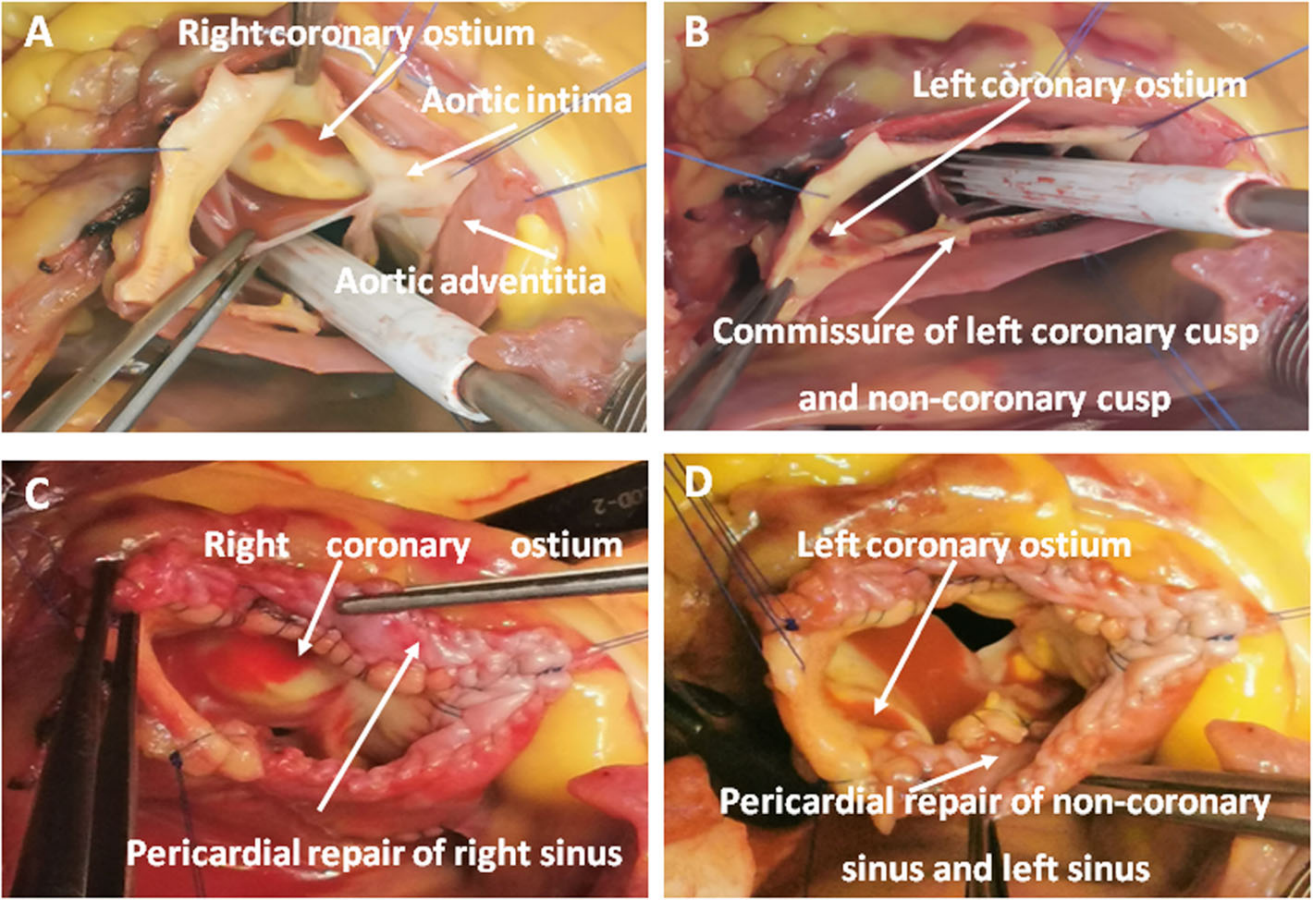
**a b** Removal of the dissection intimal flap to the normal aorta wall at the noncoronary sinus, leaving a 5 mm rim of intimal flap near the commissures and coronary ostia. **c d** Pericardial autograft patch was used to create a new aortic wall to reconstruct the sinus, coronary ostia, and resuspend the commissures. A pericardial patch was sutured to the aortic adventitia to strengthen the new aortic wall

The dissection intimal flap was removed to the normal aorta wall near the annulus at the noncoronary sinus, leaving a 5 mm rim of intimal flap near the commissures and coronary ostia (Fig. 2a, b).

Root reconstruction using pericardial autograft was initiated at the lowest point of the noncoronary sinus. The following procedure was then performed; suturing of the normal aorta near the annulus to pericardial patch, suturing the annulus and adventitia to the pericardial patch near the commissure in order to resuspend and fasten the commissure, suturing the intimal flap rim and adventitia near the coronary ostia to the pericardial patch in order to reconstruct the coronary ostia. The procedure was performed using a running 5–0 polypropylene suture. Finally, the pericardial patch and dissection adventitia was trimmed to equal height and sutured together using a running 5–0 polypropylene suture (Fig. 1b, Fig. 2c, d).

After the patient’s body temperature dropped to 25 °C, selective cerebral perfusion and deep hypothermic circulatory arrest were initiated. A frozen elephant trunk (CRONUS 260 mm × 100 mm) was inserted into the descending aorta and total arch replacement was performed using a four-branch prosthetic vessel (Terumo Gelweave 26/10/8/8_10mm). The proximal main graft of the four-branch vessel prosthesis was anastomosed to the reconstructed aortic root using a running 4–0 polypropylene suture.

The cardiopulmonary bypass time was 198 min, cross-clamp time was 128 min, and deep hypothermic circulatory arrest time was 18 min.

The patient’s recovery was uneventful. The duration of mechanical ventilation support was 19.02 h. The duration of stay in the intensive care unit was 40.15 h. The postoperative hospital stay was 12 days.

Postoperative doppler echocardiography showed trivial aortic insufficiency. A postoperative CT scan showed normal sinus morphology.

## Discussion and conclusions

There are several surgical techniques to manage acute type A aortic dissection involving the sinus. These include the sandwich and neomedia technique, sinus repair using graft patch, biologic glue fixation, and their modifications [1–4].

Root reconstruction using commissure resuspension and coronary ostia repair often leads to dissection intima and a false lumen to be remained. The residual false lumen and blood flow into the lumen from needle holes and needle hole intimal tears have the potential risk of proximal bleeding. This has adverse effects on the long-term durability of root reconstruction. A significant amount of false lumen near the coronary ostia may result in coronary stenosis due to blood flow and thrombus compression.

Using our technique, we removed almost all the intimal flap, leaving only a small amount near the coronary ostia and aortic commissures. This was performed using a pericardial patch as a new aortic wall while preserving the aortic adventitia to fix and strengthen the new pericardial aortic wall. This resulted in an increase in long-term durability and lowered the risk of proximal bleeding. This technique is suitable for the sinus diameter less than 45 mm, David procedure is used for sinus diameter more than 45 mm.

The key points of this new procedure include suturing the annulus and aortic adventitia with the pericardial patch near the commissures to resuspend and fasten the commissures. The annulus was strong, suturing the annulus wasn’t easy to form needle hole tears of intimal flap. Reconstruction of the coronary ostia by suturing the intimal flap rim and adventitia together using a pericardial patch could significantly reduce dissection tissues and remnant false lumen. This reduces the risk of coronary stenosis caused by false lumen blood flow and thrombus compression.

In conclusion, aortic root repair with pericardial autograft is a safe and effective technique to treat acute type A dissection involving the sinus. Using this technique, remnant dissection tissues could be significantly reduced, which subsequently decreases the risk of proximal bleeding and hence increases long-term durability.

## Data Availability

The datasets used and/or analysed during the current study are available from the corresponding author on reasonable request.

## Abbreviation

CT: Computed tomographic
